# Multiplex and on-site PCR detection of swine diseases based on the microfluidic chip system

**DOI:** 10.1186/s12917-021-02825-w

**Published:** 2021-03-12

**Authors:** Yan Jiang, Shan Jiang, Yue Wu, Bin Zhou, Kaimin Wang, Luyan Jiang, Yunfeng Long, Gan Chen, Dexin Zeng

**Affiliations:** 1 Animal, Plant and Food Inspection Center, Nanjing Customs, Nanjing, 210019 China; 2 MOE Joint International Research Laboratory of Animal Health and Food Safety, College of Veterinary Medicine, Nanjing Agricultural University, Nanjing, 210095 China; 3 Jinggangshan Agricultural Science and Technology Park Management Committee, Jian, 343000 China

**Keywords:** Swine disease, Microfluidic chips, PCR, Multiplex and on-site detection

## Abstract

**Background:**

At present, the process of inspection and quarantine starts with sampling at the customs port, continues with transporting the samples to the central laboratory for inspection experiments, and ends with the inspected results being fed back to the port. This process had the risks of degradation of biological samples and generation of pathogenic microorganisms and did not meet the rapid on-site detection demand because it took a rather long time. Therefore, it is urgently needed to develop a rapid and high-throughput detection assay of pathogenic microorganisms at the customs port. The aim of this study was to develop a microfluidic chip to rapidly detect swine pathogenic microorganisms with high-throughput and higher accuracy. Moreover, this chip will decrease the risk of spreading infection during transportation.

**Results:**

A series of experiments were performed to establish a microfluidic chip. The resulting data showed that the positive nucleic acid of four swine viruses were detected by using a portable and rapid microfluidic PCR system, which could achieve a on-site real-time quantitative PCR detection. Furthermore, the detection results of eight clinical samples were obtained within an hour. The lowest concentration that amplified of this microfluidic PCR detection system was as low as 1 copies/μL. The results showed that the high specificity of this chip system in disease detection played an important role in customs inspection and quarantine during customs clearance.

**Conclusion:**

The microfluidic PCR detection system established in this study could meet the requirement for rapid detection of samples at the customs port. This chip could avoid the risky process of transporting the samples from the sampling site to the testing lab, and drastically reduce the inspection cycle. Moreover, it would enable parallel inspections on one chip, which greatly raised the efficiency of inspection.

## Background

Polymerase chain reaction (PCR) is a molecular detection technology based on nucleic acid sequences [1]. After nearly 40 years development, the PCR molecular detection technology has become the primary method for detecting diseases caused by pathogenic microorganisms, and has been widely used in disease detection [2, 3], clinical testing [4, 5], food quarantine [6, 7], forensic identification [8, 9], customs inspection [10] and in various other fields [11–14]. The traditional PCR instrument is large in size and inconvenient to carry; and it has a higher demand for the experimental environmen. These drawbacks of this instrument seriously limit its scope of use, and prevent its operation in rapid on-site real-time PCR detection [15].

Porcine circovirus (PCV) belongs to the Circoviridae family, and is one of the smallest known animal viruses. The virion has no envelope, and contains a covalently closed single-strand circular negative-strand DNA. It often causes weaning piglets with multiple system failure syndrome (PMWS) and was tested using quantitative PCR and antigen capture ELISA [16]. Porcine reproductive and respiratory syndrome (PRRSV) is a member of the Arteritis virus family. According to the antigenicity, genome and pathogenicity of the virus, PRRSV can be divided into two types, namely European type and American type. It mainly causes reproductive disorders in pigs and first isolated in China in 1996 [17]. Porcine epidemic diarrhea virus (PEDV) is a single-positive-strand RNA virus, belonging to the Coronavirus. PEDV is one of the major etiologies responsible for the acute, highly contagious disease in the digestive tract of pigs, especially neonatal Piglet*s. multiplex* PCR is commonly used in clinical practice to differentiate between this virus, rotavirus and porcine epidemic diarrhea [18]. Pseudorabies virus (PRV) can cause reproductive disorders at any stage of pregnancy, piglets often show chin and respiratory symptoms. Some researchers have designed primers for gE and gB genes to establish gene chip detection methods to distinguish between field and vaccine virus [19].

The microfluidic chip [20–22], also known as lab on a chip, combines and arranges various unit technologies on a small platform to implement all the biochemical laboratory experiments including sampling, sample introduction, sample preparation, reaction, product testing, and so on [23, 24]. Compared with the traditional PCR method, the microfluidic chip is much smaller in size and more portable along with much less reagents. It, changed temperature more rapidly, integrated with other devices more easily, and had more moderate demands for experimental environment and lab assistants [25–28]. All these advantages brought the microfluidic chip into the focus of research on rapid on-site real-time PCR detection [29–32]. Pork and its processed products, as a traditional food, have become an indispensable part in people’s diet [33]. China Customs, as the only pass for import and export of goods and commodities into and out of the country, is responsible for the inspection and quarantine of tens of thousands of pigs, and large amounts of pork and its processed products; which calls for rapid, efficient and safe work [34]. The current detection methods cannot meet the needs of rapid detection [35, 36]. Therefore, development of a technology for rapid and high-throughput detection of pathogenic microorganisms at the customs port is of great significance. This study was to develop a microfluidic chip to be applied to rapid and high-throughput swine disease detection with higher accuracy and lower risk of spreading pathogenic microorganisms during transportation.

## Results

### The lowest amplification concentration of PCR microfluidic chip assay

**Fig. 1:**
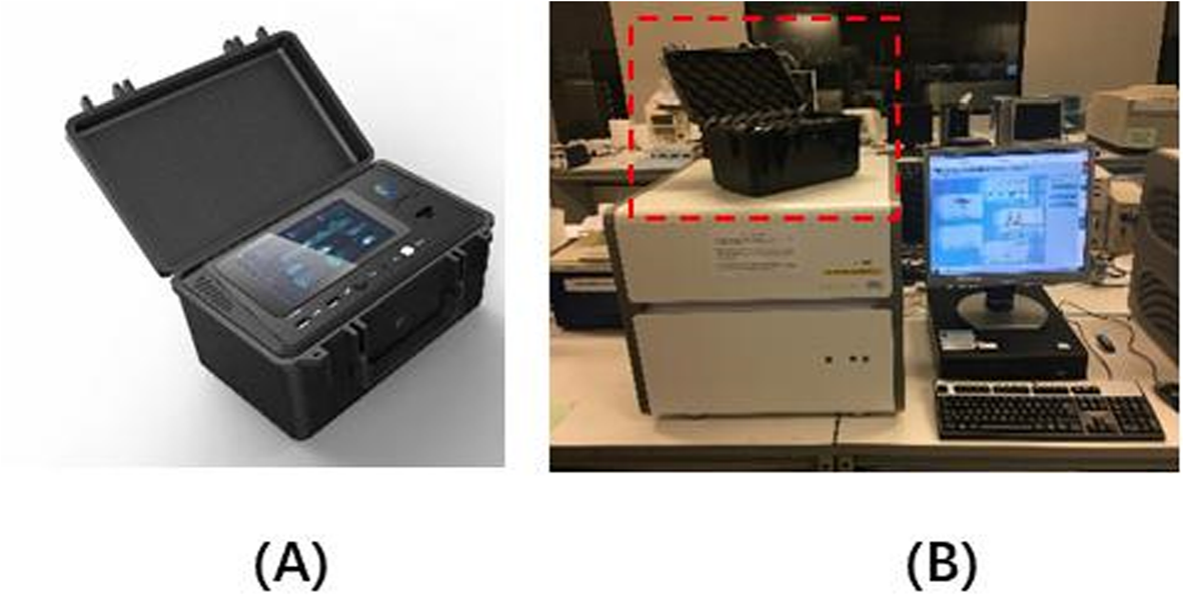
The lowest amplification concentration of the PCR protocols of the four swine disease viruses

We chose four principal swine viruses, including PRRSV, PEDV, PRV and PCV2, which are often found in related inspection at the customs, and we synthesized the plasmids of these viruses as positive references. The concentrations of the primers and probes, and the reaction temperatures were optimized by means of the plasmid amplifications on the PCR microfluidic chips. The microfluidic chip had 8 chambers, and each chamber accommodate 2 μL of the PCR reaction solution. The volume of the amplification system was reduced that was helpful in the rapid PCR process. The rapid heat transfer kept the activity of the Taq DNA polymerase at the maximum level during the rise and fall of temperature. The microfluidic chip with silicon as its substrate further accelerated the heat transfer during the PCR thermal cycle, because of silicon’s excellent thermal conductivity. It was obvious that the amplification reaction’s fluorescence intensity on the chip presented itself in a typical S-shaped curve, and the negative control chamber, in which sterilized water was used as the template, did not show any amplification, indicating that the specificity of the primers and the probe, and the optimization of the amplification temperature were all appropriate here. The initial concentration of the four plasmids was 10^9^ copies/μL. All the plasmids were 10-fold serial diluted to the final concentration of 1 copies/μL, and every concentration was tested. As shown in Fig. 1, the lowest concentration that amplified of the four positive plasmids were 10 copies/μL for PRRSV, 10 copies/μL for PEDV, 100 copies/μL for PRV and 1 copy/μL for PCV2, respectively, which would meet the requirement for detection in terms of the virus load obtained from the clinical samples of the animals.

In another experiment, nucleic acid extracted from 10-fold serial dilutions of viruses of known titre was used to determine the minimum detectable quantity of the PCR microfluidic chip assay for each virus. As shown in Table 1, the PCR microfluidic chip assay was most efficient for detection of RPV and had a minimum detectable quantity of 1 TCID_50_/mL.

**Table 1:** Limit of detection of PCR microfluidic chip assay and real time PCR assay

Virus	Real time PCR assay(TCID_50_/mL)	PCR microfluidic chip (TCID_50_/mL)
PRRSV	1 × 10^2^	1 × 10^3^
PEDV	1 × 10^3^	1 × 10^3^
PRV	1 × 10^0^	1 × 10^0^
PVC2	1 × 10^2^	1 × 10^2^

### The specificity testing using positive nucleic acid

**Fig. 2:**
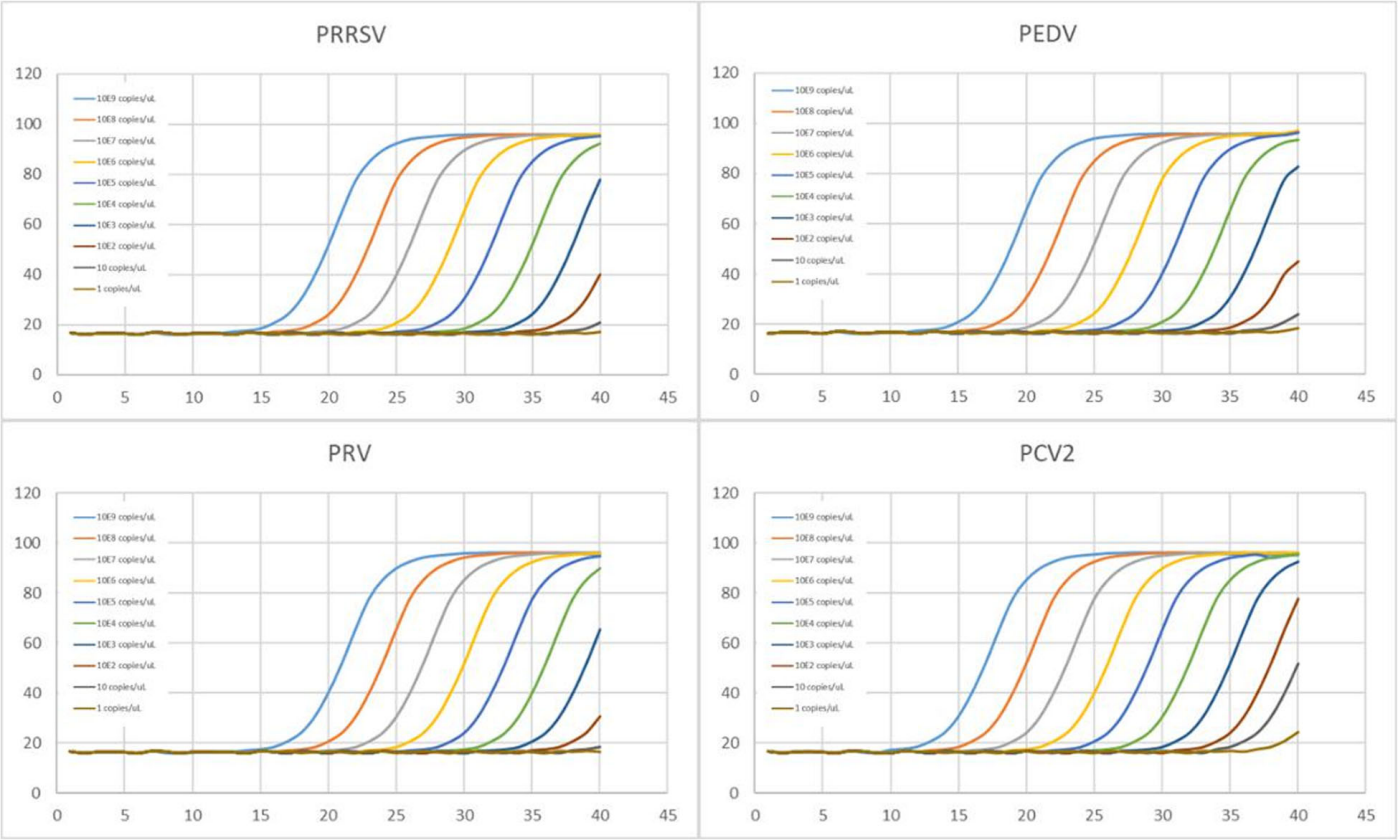
Specificity test of the four swine disease viruses on the chip

Besides the sensitivity test, we also verified the specificity performance of the PCR detections on the microfluidic chip. Based on the positive nucleic acids of four viruses isolated from the clinical sick pigs, which performed the typical symptoms of reproductive disorders, multi-system failure of weaned piglets, and watery diarrhea. We chose one sample as the target for the specificity test, and the other three samples were used as the control. The PCR amplifications were performed on one chip with one reaction chamber filled with the target sample, the rest of the chambers filled with the other three samples as well as other two porcine-transmitted viral agents including porcine transmissible gastroenteritis virus (TGEV) and procine rotavirus (PRVA). As shown in Fig. 2, the result showed that the four PCR protocols all had a very high specificity, because only the target sample presented a significant amplification curve and the control samples had no amplifications. The fast amplification process of the PCR system contributed not only to the high specificity of the PCR amplification but also the short duration in the non-required temperature zone.

### The detection performance of the microfluidic PCR system using positive nucleic acid

**Fig. 3:**
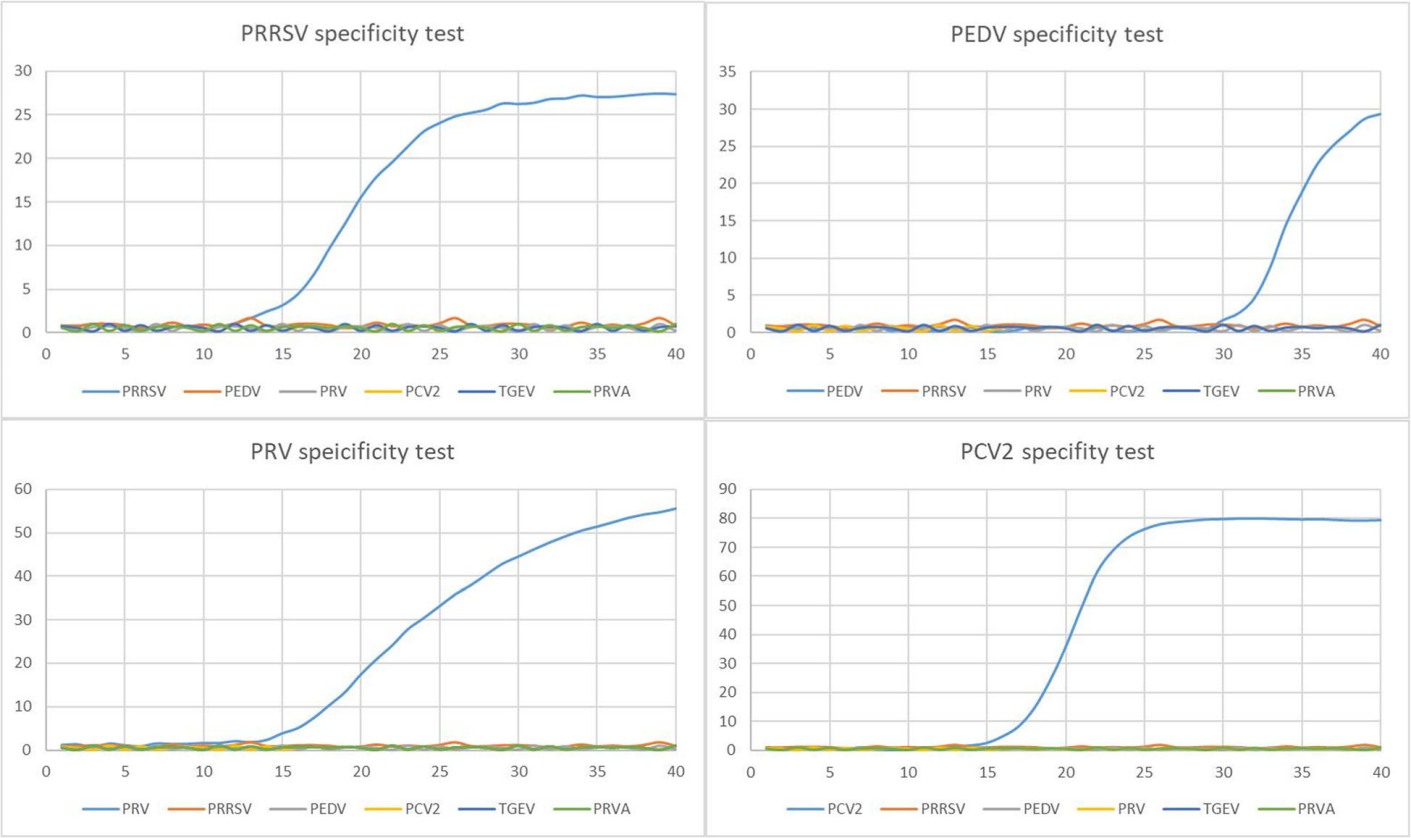
Parallel detection results of four clinical samples of swine diseases on the 8-chamber microfluidic chip (**a**) and on traditional tube-based PCR instrument (**b**)

Finally, we verified the PCR microfluidic chip detection performance using the positive nucleic acid of swine pathogens. Every two chambers on the chip were used for the reaction of one clinical sample, and one chip accommodated four samples in parallel reactions. At the same time, we also performed the detection for the viruses of four swine diseases on a traditional tube-based PCR instrument for parallel reference. As shown in Fig. 3a, each of the four samples had a significant S curve with a varied Ct value (17.5 for PRRSV, 33.5 for PEDV, 18.5 for PRV and 16.5 for PCV2, respectively). The Ct values of the four swine diseases tested on chip-based and tube-based PCR platform showed no significant differences (Fig.3b). The differences in the Ct values were due to the variety in the virus abundance of each of the original positive nucleic acid. The magnitude of the fluorescence intensity increment in the different positive nucleic acid at the end of the reaction was due to the different amplification efficiency of primers and probes for different target gene sequences. However, the Ct value was usually used as a criterion when determining the positive or negative nature of the sample. The sample with a Ct value smaller than 35 could be considered as positive threshold, and the sample with a Ct value greater than 35 needed to be re-examined.

## Discussion

The aim of this study is to develop a fast and on-site swine disease detection solutions to decrease the transit time for customs clearance. Point of care testing could be the optimal choice for the on-site detection. Compared with molecule hybridization [36], LAMP [37] and other isothermal amplification technology, PCR technology as the gold standard for infectious disease detection which has been widely applied. However, the current PCR performance still need sample transport to testing lab and the resulting data could not been got in less than 3 h [38]. The challenge is that the sample have a risk of degradation if the storage condition is not proper as well as extra cost from the transportation. Therefore, a portable and miniaturized PCR detection platform is needed to develop. Herein, the microfluidics chip based PCR testing solution along with short testing duration, multiple testing capability and instrument portability was established for diagnostic assay.

Compared to traditional lab-based PCR detection that must be finished in the central lab, our portable PCR tester could realize an on-site detection for swine diseases and a rapid result observation. In addition, four swine pathogens could be detected on a single chip simultaneously. A rapid thermal cycling capability of the PCR tester with the help of microheater realized a shorter detection time, which is helpful for a fast custom clearance and also for an efficient disease control to decrease the further spread risk of infection disease. In order to realize an on-site molecular test, many factors need to be considered, including testing duration, instrument portability, operation complication, contamination and so on. A real time PCR method may avoid the contamination of this process and need no more offline analysis because of a miniaturization of the optical detection module for a fluorescent detection. The main consideration in the design of the experimental scheme is to focus on the establishment of the amplification detection system of the PCR microfluidic chip, so performing a rigorous study of the sensitivity of the method is outside the scope of the present work. We just determined the lowest amplification concentration of PCR microfluidic chip assay by using the standard plasmid and viruses of known titre. As for the specificity of this method, no cross reaction occurred when more than one PCR detection performed in a single chip well, suggesting that the PCR reagent component had a good compatibility with the chip surface. Additionally, a fast thermal cycling could increase the PCR enzyme amplification efficiency. Therefore, this chip is helpful for the further development of a multiple optical channel instrument for high-throughput multiplex PCR detection. In the future, a more high-throughput chip that could realize a more than 16 samples testing on a single chip will be developed, which could accommodate more samples for simultaneous detection, along with higher throughput, greater efficiency and accuracy. The further study also needs to develop an automated sample preparation system for nucleic acid extraction and purification, which will decrease the on-the-spot detection duration [39–41].

## Conclusion

In this study, An on-site microfluidic PCR detection system established meeted the requirement for rapid detection of samples at the customs port. All the detection were completed within 1 h. Application of this chip would effectively help the on-site inspection to be carried out and it could avoid the risky process of transporting the samples from the sampling site to the testing lab and drastically reduce the inspection cycle.

## Methods

### Reagents and equipment

The magnetic bead virus nucleic acid extraction kit was purchased from Suzhou Tianlong Bio-Technology Co. Ltd. The reverse transcriptase was from Vazyme Biotech Co. Ltd. (Nanjing), and the Taq DNA polymerase was from Fapon Biotech Inc. (Zhuhai). The Power SYBR Green PCR Master Mix Kit was from ABI Co.Ltd. (USA)*. O*neStep ABI instrument (USA). The primers and probes used in common epidemic swine diseases detection were synthesized by Genscript (Nanjing) Co., Ltd., and the detailed sequences are shown in Appendix Table 2. The microfluidic PCR analyzer was purchased from Shenzhen Shineway Technology Corporation and the fluorescence quantitative PCR instrument was from Hangzhou Bioer Technology Co. Ltd.

### Nucleic acid extraction and amplification

The nucleic acid of the pathogenic microorganisms were extracted following the instructions of the magnetic bead nucleic acid extraction kit. For synthesis of cDNA, 16 μL master mix consisted of RT-PCR buffer, dNTPs, random hexamer, reverse transcriptase enzyme and RNase inhibitor was mixed with 24 μL of extracted RNA. Reverse transcripyion was performed at 37 °C for 1 h. The extracted DNA solution was used as the template solution for the subsequent experiments, and was stored at − 20 °C in a refrigerator; or at 4 °C in a refrigerator or on ice in case of immediate use in a subsequent experiment. In the nucleic acid amplification, a 25 μLreaction solution was prepared with the following components: 5 μLtemplate solution, 0.5 μLforward primer (10 μM), 0.5 μLreverse primer (10 μM), 0.2 μLprobe (10 μM), 0.2 μLreverse transcriptase, 0.5 μLTaq DNA polymerase, 5 μL5X reaction buffer, and 13.1 μLsterilized water. All these components were fully mixed in a PCR tube and added to the microfluidic chip by a pipette, then PCR amplification was performed. The process of the amplification included Step One: reverse transcription at 50 °C for 15 min; Step Two: denaturation at 95 °C for 3 min; and Step Three: 40 cycles at 95 °C for 15 s and at 55 °C for 45 s.

### Real-time PCR conditions

DNA or cDNA was amplified by a real-time PCR using Power SYBR Green PCR Master Mix Kit in OneStep ABI instrument. Each reaction had a total volume of 25 μL, including 12.5 μL SYBR Green master mix, 200 nmol of each forward and reverse primers, 5 μL cDNA plus 7.1 μL ddH2O. The cycling conditions included an initial denaturation step of 10 min at 94 °C, followed by 40 cycles of 15 s at 95 °C, 1 min at 55 °C and 1 min at 60 °C. Fluorescent detection was at the end of each cycle. Melting curve analysis program was used for identification of specific PCR products.

### Chip design and preparation

There were 8 reaction chambers with a volume of 2 μL each on the PCR microfluidic chip. The PCR microfluidic chips were made of silicon wafers of 500 μm thick with both sides polished and heat-resistant glass substrates of 500 μm thick by Shenzhen Shineway Technology Corporation. The PCR reaction chambers and channels on the silicon wafers were formed by dry etching using a semiconductor photolithography process. Then, sixteen holes were drilled on the silicon substrate which served as the inlets and outlets of PCR solutions, respectively. Finally, the silicon wafers were bonded to the glass substrates and the two-layer wafers were cut by a dicing saw to form independent PCR detection chips.

A standardized semiconductor fabrication process was utilized to ensure the yield rate and uniformity in quality of the chips. And the automated fabrication process could significantly reduce the cost of each single inspection by expanding the size of the silicon wafers.

### The amplification detection system of the PCR microfluidic chip

**Fig. 4:**
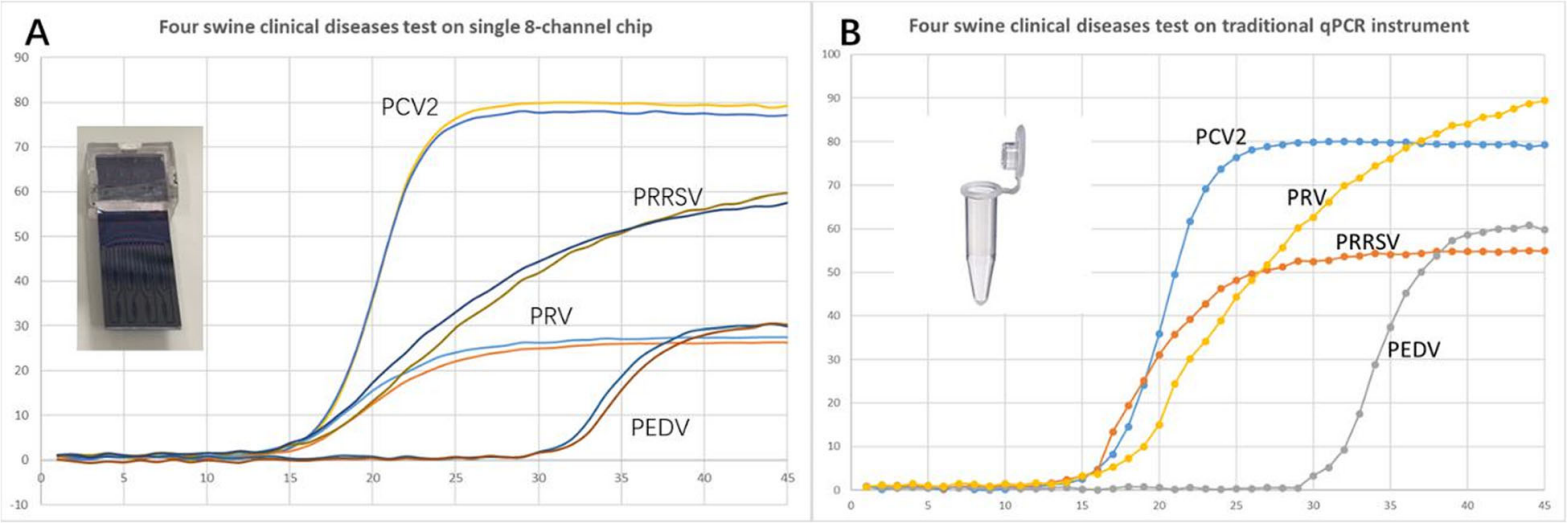
**a** The portable rapid microfluidic PCR system established in the lab of Dr. Yan Jiang; **b** Size comparison of the microfluidic PCR instrument (within the red dotted box) with the traditional large PCR instrument (Roche 480)

A portable rapid microfluidic PCR system (SWA-01, Shenzhen Shineway Technology Corporation) was used to perform the real-time PCR amplification of the samples (Fig. 4). The dimension was 330 mm(L) × 320 mm(W) × 160 mm(H)and the weight was round 3 kg, suggesting it had a small footprint. Used in conjunction with the specially supplied reagents and the microfluidic chips, the SWA-01 PCR instrument is applicable to rapid real-time fluorescence detection of PCR in the fields of animal quarantine, food safety and public health and safety. The portable rapid microfluidic PCR instrument was mainly composed of a control system (with a touch screen graphic user interface), a power supply system, a photoelectric system, a temperature control system [35], an outer jacket part, a microfluidic chip, and a software module. The instrument had the following advantages for on-site detection as follows: portable design, low weight, rapid thermal cycling and real time detection.

## Data Availability

Raw data is available from the corresponding author on reasonable request.

## Appendix

**Table 2:** The sequences of the primers and probes of the four swine diseases

The amplification gene.	5′ → 3′ Sequence
PRRSV-ORF1b gene	F1: GCCTGACTAAGGAGCAGTGTTTA
	R2: ACCGCTGTCTCAGTAACAACTAAGC
	P3: CCGCGACCACAGCGGGTCA
PCV2-ORF1 gene	F: TGGAGAGCGGGAGTCTGGT
	R: CTTCCAATCACGCTTCTGCAT
	P: ACCGTTGCAGAGCAGCACCCTGT
PEDV-Spike protein gene	F: TCGCAATCTCAGCGTTATGGT
	R: GGTGCTGCCTGTACCAGAGAGA
	P: TGTGGTGGTGATGGCGAGCACAT
PRV-E (gE) gene	F: TTGTGCTGGCGCTGGGCTCCT
	R: CGACGCGATCTACGTGGAC
	P: CACAGCACGCAGAGCCAGA

*F* the forward primer, *R* the reverse primer, *P* the probe

## Abbreviations

PCR: Polymerase chain reaction;
ELISA: Enzyme-linked immunosorbent assay
LAMP: Loop-mediated isothermal amplification
